# COVID-19 Deaths in Transnational Settings: Disrupted Bereavement and Pandemic-Related Prolonged Grief Disorder in the Latinx Immigrant Population

**DOI:** 10.1177/15404153241290175

**Published:** 2024-10-10

**Authors:** Kelsey Shaulis, Victor Garcia

**Affiliations:** 1 Department of Sociology, 8082 The Pennsylvania State University, University Park, PA, USA; 2 Senior Research Scientist, 14652 Prevention Research Center, Berkeley, CA, USA

**Keywords:** grief, bereavement, mental health, immigrant health, COVID-19

## Abstract

The Latinx immigrant population experienced one of the highest COVID-19 death rates. Those left behind have exhibited rising rates of mental illness, particularly, pandemic-related prolonged grief disorder. The Latinx immigrant population is uniquely vulnerable to this disorder as a result of disrupted culturally appropriate bereavement practices, constrained social support, and concurrent COVID-19 stressors and immigration-related trauma. Despite a rising call for research on pandemic-related prolonged grief disorder, little is known about the true prevalence, cause, and appropriate treatment protocol behind this disorder in the Latinx immigrant population. Four areas of research critical to the identification and understanding of pandemic-related prolonged grief disorder in this population are recommended: 1) death and bereavement in transnational settings, 2) immigrant social networks and disrupted bereavement, 3) COVID-19 stressors and grieving, and 4) prolonged grief disorder and mental illness comorbidities. An understanding of these four contributing areas to Latinx immigrants’ vulnerability to prolonged grief disorder is imperative to providers’ development of assessments and treatment protocols needed to identify and treat prolonged grief disorder in this population.

Four years have passed since the SARS-CoV-2 virus (henceforth COVID-19) was declared a global pandemic. Increasingly, concern about the virus is winding down in the general population, and many are looking to put the pandemic behind them. Moving on will be easier for some than for others. For the Latinx population, it will be a challenge, especially for those who continue to struggle with untreated prolonged grieving and compounding mental illnesses. They experienced one of the highest COVID-19 death rates and historically suffer from severe health disparities and inequitable access to care (Brenes, 2020; Diaz et al., 2023; NCHS, 2023; Ormiston et al., 2023).

Despite the call for research on pandemic-related prolonged grief disorder (PGD) (Brenes, 2020; Eisma & Boelen, 2023; Ormiston et al., 2023), little is known about the prevalence and causes behind this disorder in the Latinx immigrant population and its post-pandemic impact on mental health. According to the DSM-5, this disorder extends beyond a year after the loss of a loved one, does not conform to cultural and religious grieving norms, affects the bereaved's mental and physical health, and interferes with interpersonal relations and daily responsibilities (APA, 2022; Szuhany et al., 2021). There is evidence that Latinx immigrants are at an increased risk for this disorder because of disrupted culturally appropriate bereavement practices and constrained social support (Falzarano et al., 2022; Simon et al., 2020). COVID-19 stressors and pre-existing mental illness disorders may also contribute to PGD onset and severity (Amy & Doka, 2021; Eisma et al., 2020; Eisma & Boelen, 2023; Garcini et al., 2024). There is a need to examine and understand the vulnerability of Latinx immigrants to PGD and the current prevalence rate in this population. Only then can researchers and practitioners develop evidence-based assessments and therapies for Latinx immigrants suffering from the disorder. Four related research areas are recommended to understand how disrupted bereavement practices and diminished social supports may have contributed to PGD among Latinx immigrants: 1) death and bereavement in transnational settings, 2) immigrant social networks and disrupted bereavement, 3) COVID-19 stressors and grieving, and 4) PGD and mental illness comorbidities.

## **U.**S**.** Latinx Population and the COVID-19 Mortality Toll

The size of the Latinx immigrant population during the pandemic was approximately 20 million (Haner & Lopez, 2023). Consistently, it has remained one of the youngest racial-ethnic groups in the U.S. (Schaeffer, 2019). When adjusted for age, the Latinx population experienced a disproportionately large percentage of COVID-19 deaths in all age groups (NCHS, 2023). Immigrants, particularly the unauthorized, likely composed a large portion of these COVID-19 deaths. Many were classed as “essential workers” and worked in unprotected conditions exposing them to the virus (Grooms et al., 2022; Olayo-Méndez et al., 2021; Salinas & Salinas, 2022). Also, economic need, reliance on public transportation, and overcrowded housing conditions inhibited the ability to socially distance (Gonzalez et al., 2021; Moyce et al., 2021; Rodriguez et al., 2021; Vargas & Sanchez, 2020). In multigenerational households, multiple family members contracted the virus, and substantial risk for hospitalization and mortality was experienced by elderly family members (Calvo & Waters, 2023; Clark et al., 2020). These pandemic conditions contribute to a large population of bereaved community members facing concurrent pandemic stressors.

## Pandemic-Related Mental Illness and Disrupted Bereavement

Across the country, mental illness increased substantially during the COVID-19 pandemic (Blanchflower & Bryson, 2022; Ettman et al., 2020; Ormiston et al., 2023). The Latinx adult population had “higher prevalence of anxiety or depressive disorder, trauma-related and stressor-related disorder, substance use to cope with stress, and suicidal ideation” in comparison to non-Hispanic White adults (Czeisler, 2020). And the disruption of bereavement has been identified as a major stressor and associated with greater grief severity (Carson et al., 2023; Lee et al., 2021), including PGD (Eisma et al., 2020; Eisma & Boelen, 2023; Garcia, 2022). Notably, non-citizen and unauthorized Latinx immigrants experienced an exacerbation in mental illness symptoms associated with their unique position in the U.S. Fears of deportation, exclusion from social programs, and disproportionate exposure to COVID-19 deaths exacerbated the risk of pandemic-related mental illness (Brenes, 2020; Goldman-Mellor et al., 2023; Pineros-Leano et al., 2022). Despite the need, mental health care was difficult to access because of persistent structural and systemic barriers, irrespective of immigration status (Blackburn & Sierra, 2021; Galvan et al., 2021; Hill et al., 2021).

## Transnational Settings and Pandemic-Related Bereavement Research Recommendations

The literature on bereavement in the Latinx population, except for a few studies (e.g., Bravo, 2017; Falzarano et al., 2022; Hidalgo et al., 2021), overlooks immigrants’ transnational culture, defined as a culture with influences from both their homelands and U.S. communities (Chavez, 2020; Viruell-Fuentes & Schulz, 2009). This limits our insights on the relationship between disrupted bereavement and PGD in this population. Latinx immigrants build community in the U.S. without severing ties to their homeland. This connection has created transnational settings (Garcia, 2013; Soehl & Waldinger, 2010), where immigrants experience life and death in more than one country. Death, burials, bereavement, and their relationship to mental illness, consequently, must be examined in this context.

### Death and Bereavement in Transnational Settings

Studies (e.g., Bravo, 2017; Falzarano et al., 2022; Hidalgo et al., 2021) show that Latinx immigrant burials in the U.S. follow the culture, customs, and religious beliefs from the immigrant's home country. However, to what degree this is the case and under what circumstances was not examined. Traditional homeland burials may not always be possible in the U.S. because of limited family economic resources, constricted kin networks, and funeral regulations. Latinx immigrant burials need to be studied according to their immigrant and cultural contexts to understand how bereavement was disrupted during the COVID-19 pandemic. Burials and their rituals demarcate important mourning phases in the culture, and to comprehend them they need to be studied together. Bereavement in traditional Latinx culture does not start and end with death. For example, it begins when the family learns of the inevitable death of a loved one at which time the family provides palliative care and prepares itself for the loss (Adames et al., 2014; Ko et al., 2017; O’Mara & Zborovskaya, 2016), and continues with post-burial activities, such as memorials, multi-day observances, and annual masses in honor of the deceased (Cann, 2016; Diaz-Cabello, 2004).

Latinx immigrants may have been particularly susceptible to the disruption of bereavement resulting from COVID-19 pandemic, whether experiencing the death of a loved one in the U.S. or their home country or a repatriated burial. Mourning phases in the U.S. were disrupted as hospitals restricted visitors, social distancing prevented gatherings, and delays in releasing bodies interrupted the bereavement process for weeks (Burrell & Selman, 2022; Chen, 2022; Eguiluz et al., 2022; Routen et al., 2021). For transnational deaths of relatives in the homeland and repatriated burials, bereavement was complicated, if not prohibited, by strict pandemic travel restrictions, irrespective of immigration status (Bravo, 2017; Garcia, 2022; Garcini et al., 2020; Lipscomb & Salinas, 2020). This inability to participate in transnational bereavement has been associated with prolonged grieving (Bravo, 2017; Garcia, 2022; Garcini et al., 2020), and may contribute to the development of pandemic-related PGD alongside concurrent COVID-19 stressors.

### Immigrant Social Networks and Disrupted Bereavement

The literature on Latinx immigrants (e.g., Ayón & Naddy, 2013; Benavides et al., 2021; Held, 2018) has documented the importance of social networks in the immigration process. Through these networks, information, resources, and culture flow across national borders, and provide a sense of community and support for immigrants in the U.S. Social networks are also a major source of emotional support during a crisis (Dozi & Valdivia, 2011; Hurtado-de-Mendoza et al., 2016; Ruiz-Sánchez et al., 2021), but their role in the bereavement process has not received the same research attention. During the pandemic, social networks were unable to provide support for families grieving a death. Social distancing measures did not permit attending burials, wakes, and visiting the bereaved afterwards (Burrell & Selman, 2022; Rivera Rivera et al., 2021). Virtual contact alternatives may have been unavailable due to prohibitive entry levels of user knowledge and connectivity availability and costs, especially among immigrants already facing economic insecurity (Fox & Garcia, 2021; Qian et al., 2022). Consequently, immigrant families could not fall back on traditional bereavement practices, and worse yet suffered from social isolation during a time of need. Isolation made culturally appropriate bereavement difficult, if not impossible, and the bereaved were susceptible to PGD that may have gone undiagnosed and untreated because of the unavailability of mental health care.

### COVID-19 Stressors and Grieving

The impact of COVID-19 stressors on the Latinx population also needs additional study, especially considering the constricted social networks during the pandemic. Latinx immigrants disproportionately suffered from COVID-19 stressors that made grieving worse and potentially resulted in PGD, among them, fear of contracting the virus, social isolation, and economic uncertainty. They were systemically more prone to these stressors than the general population (Garcia et al., 2021; Gauthier et al., 2021; Vargas & Sanchez, 2020). Contracting the virus was a likely possibility for immigrants who were “essential” workers, as was contaminating others at home in overcrowded multigenerational households. And some immigrants lived in constant fear of losing their jobs, as industries, like the restaurant and service industry, were closing and going out of business. These stressors led to intense emotional distress (Czeisler, 2020; Moon, 2021) and disrupted the bereavement practices of those who lost a loved one, particularly when death rates were high early in the pandemic (Burrell & Selman, 2022; Chen, 2022; Eguiluz et al., 2022). Over time, they, too, could have resulted in a PGD, and together with other untreated pre-pandemic stressors made immigrants vulnerable to mental illness comorbidities.

### PGD and Mental Illness Comorbidities

According to the APA, individuals with PGD often experience mental illness comorbidities (APA, 2022), among them, post-traumatic stress disorder (PTSD). PTSD is highly prevalent in the Latinx immigrant population, and the literature is replete with evidence of this trauma with origins in symbolic, structural, and physical violence. Immigrants are subject to violence in their homelands, along their journey north, and throughout their time in the U.S. (Clemens, 2021; Enríquez, 2017; Estefan et al., 2017; García et al., 2022; Hurtado-de-Mendoza et al., 2016; Leyva-Flores et al., 2019; Valdivia Ramírez, 2022). Disproportionately women and those with non-heterosexual identities are targeted (Alessi et al., 2021; Chavez, 2020; Cleaveland & Frankenfeld, 2020; Enríquez, 2017). Because of mental health care barriers in the immigrant community, many do not receive care for PTSD or ongoing violence. The costs can be prohibitive, the availability limited, and mental health providers with expertise in this kind of trauma scarce. Additionally, depending on location, unauthorized immigrants may not qualify for mental health care, unless they reside in a state with immigrant-inclusive state-funded programs. PTSD, if left untreated, may aggravate PGD together with other mental illness disorders (Derr, 2016; Grafft et al., 2022; Kaltman et al., 2010).

## Conclusion

For some time now, there has been a call for public health to improve mental health services for the Latinx immigrant population (Ayón et al., 2020; Calzada et al., 2020; Derr, 2016), and lately it includes PGD with origins in the pandemic (Brenes, 2020; Villatoro et al., 2022). Knowledge of the transnational setting of Latinx immigrants, especially when it comes to death and bereavement, social networks, COVID-19 stressors, and immigration-related PTSD can inform mental health practitioners about the complex causes behind pandemic-related PGD. More importantly, it can also support the development of assessments and specialized mental health care for immigrants suffering from PGD and other concurrent mental illness disorders. Assessments can assist practitioners in identifying these disorders and monitoring and determining the effectiveness of treatment protocols. They can also assist mental health care providers to provide comprehensive and informed culturally competent PGD treatment designed for Latinx immigrants. Only by achieving this, will providers be able to identify and treat PGD and comorbid long-term mental illnesses identified as contributors to the rapid decline in the general mental health in this population (e.g., Brenes, 2020; Villatoro et al., 2022; Snowden et al., 2023).
